# Behavioural activation for people in custody with depression: A protocol for a feasibility randomised controlled study

**DOI:** 10.1371/journal.pone.0304767

**Published:** 2024-06-13

**Authors:** Sandra M. Walsh, Kuda Muyambi, Shaun Dennis, Steven Hutchinson, Tom Turnbull, Kuan Liung Tan, Pascale Dettwiller, Daniel Bressington, Richard Gray, Lucy Howard, Joanne Andrews, Shyamsundar Muthuramalingam, Vincent L. Versace, Martin F. Jones

**Affiliations:** 1 University of South Australia Department of Rural Health, Whyalla Norrie, South Australia, Australia; 2 University of South Australia IIMPACT in Health, Adelaide, South Australia, Australia; 3 Flinders and Upper North Local Health Network, Whyalla Integrated Mental Health Service, Whyalla, South Australia, Australia; 4 South Australia Prison Health Service, Central Adelaide Local Health Network, South Australia, Australia; 5 Faculty of Nursing, Charles Darwin University, Casuarina, Northern Territory, Australia; 6 Faculty of Nursing, Chiang Mai University, Chiang Mai, Thailand; 7 La Trobe University, Bundoora, Victoria, Australia; 8 Faculty of Health, Deakin Rural Health, School of Medicine, Deakin University, Warrnambool, Victoria, Australia; NYU Grossman School of Medicine: New York University School of Medicine, UNITED STATES

## Abstract

People in custody are at high risk of developing depression. Accessing psychological treatments in a prison setting is a particular challenge, in part, due to difficulties accessing specialist mental health workers. Behavioural Activation (BA) may be helpful in improving health outcomes for people in custody experiencing depressive symptoms. The aim of this study is to establish the feasibility and acceptability of custodial health nurses delivering BA to improve depressive symptoms of people in custody. We will conduct a pilot randomised controlled trial with process observation examining the feasibility and acceptability of BA in treating people in custody with depressive symptoms. 60 people in custody presenting with depressive symptoms will be randomised to receive BA plus treatment as usual (TAU) or TAU provided by custodial health nurses. Eight custodial health nurses will be recruited, trained, and deliver BA. BA will be delivered twice a week for six weeks, with sessions lasting up to 30 minutes. Changes in depression and quality of life (QoL) will be assessed at baseline, 6 weeks, and 3 months post-intervention. Participants will be interviewed to understand feasibility and acceptability of BA in prison settings. The findings will inform the design of a randomised controlled trial to test the efficacy of BA for people in custody with depression. Findings will help determine whether BA for depression is suited to prison health care system and services. Improving depressive symptoms in people in custody has benefits beyond prison settings. The Central Adelaide Local Health Network Human Research Ethics Committee and University of South Australia Human Research Ethics Committee have approved the study. The trial results will be disseminated through peer-reviewed journals and scientific conferences and reported to local stakeholders and policy makers. If feasibility and acceptability is demonstrated, we will seek to progress to an effectiveness study. A potential strength of the trial model proposed, is in its scalability, with potential to increase the trial sites and locations. This trial has been prospectively registered with the Australian New Zealand Clinical Trials Registry (reference number: ACTRN12623000346673p).

**Trial registration** ACTRN12623000346673p.

## Introduction

People in custody are at an increased risk of developing mental health problems, particularly depression [1–3]. In a systematic review of 109 studies involving 33, 588 people in custody, conducted in 24 countries during the period 1966 to 2010, the authors reported a pooled prevalence of major depression of 10.2% in males and 14.1% in females in custody [4]. This is more than double the estimated prevalence of depression in the general population (4% men, 6% women) [5]. Considering that approximately 10 million people are in prison worldwide [6], the burden of depression amongst people in custody may represent an issue of public health concern. Depression is often underdiagnosed and under-treated in prison settings [1]. Treating depression in people in custody can have several economic and social benefits. For instance, treating depression in people in custody reduces the risks of suicide [7] and self-harm [8] whilst in custody, and premature mortality [9], recidivism [10], and violence and victimisation [1] on release from prison.

The recommended treatments for depression include antidepressant medication and psychotherapy used singularly or in combination [11]. However, the use of antidepressants in custodial settings is problematic with issues concerning medication adherence, misuse, and adverse effects [12]. Cognitive behavioural therapy (CBT) is the recommended treatment for depression in adults [11]. In a systematic review of 37 randomised clinical trials (RCTs) undertaken by Yoon et al. [2017], CBT is modestly effective in treating depression and anxiety in people in custody [13]. However, accessing CBT in prison healthcare environments can be difficult. Typically, one needs to be a specialist mental health worker (for example, psychologist or mental health nurse) to deliver CBT. Accessing these types of specialist mental health workers in prison can be challenging.

A simpler alternative to CBT is BA, which teaches people to monitor fluctuations in their mood and identify the behaviours they were engaging in at the time. Activities which have a positive effect on mood are then scheduled to increase these activities while minimising those that are less beneficial [14,15]. A comparison of the effectiveness of CBT and BA in the treatment of depression indicated that both therapies were equally effective [16]. A strength of BA is that you do not need to be a specialist mental health worker or trained health care worker to deliver BA. Preparing workers to practice BA is relatively short [16], 5–10 days, compared to CBT which takes up to 18 months. The theoretical underpinning of BA is that people improve their mood if they routinely engaged in pleasurable events and minimise avoidance [14,15].

This clinical trial will investigate the feasibility and acceptability of custodial health nurses delivering BA to support people in custody with depression. Feasibility trials are important for determining whether aspects of a study will work and to estimate the parameters, such as sample size and potential attrition rates, for a larger trial should one be conducted [17]. By conducting a feasibility trial, we are also able to consider whether BA, as an intervention, is an acceptable intervention in a prison environment. This includes whether custodial nurses and people in custody, consider nurse-deliver BA in the prison environment acceptable. If this trial demonstrates feasibility and acceptability, a larger trial would be warranted, and the intervention may help to increase access to a simple, cost-effective, and evidence-based psychological approach.

### Aim

We will examine the feasibility and acceptability of custodial health nurses delivering BA plus usual SA prison health care (intervention) compared to usual SA prison health care alone (treatment as usual (TAU)) in the management of depression in people in custody who experience depressive symptoms.

### Objectives

#### Custodial health nurses

1. Establish the proportion of custodial health nurses who complete online BA training and are assessed as being competent.
2. Establish custodial health nurses’ fidelity to BA treatment.
3. Explore custodial health nurses’ experiences of BA training and supervision via telehealth and delivering BA treatment to people in custody supported by telehealth.

### People in custody

1. Establish the number of people in custody accessing SA prison health services who report experiencing clinically meaningful depressive symptoms.
2. Determine the number of people in custody with clinically meaningful depressive symptoms who are approached and agree to participate in the trial.
3. Determine the proportion of eligible people in custody who have agreed to participate and agree to be randomised.
4. Determine the proportion of people in custody with clinically meaningful depressive symptoms that participate in the trial who complete baseline measures.
5. Determine the proportion of people in custody who complete a BA treatment (attending for a minimum of six sessions over six weeks)
6. Determine the proportion of people in custody who complete outcome measures at six weeks and three-month follow-up.
7. Calculate the preliminary efficacy of BA on depressive symptoms (primary outcome) and improving health-related quality of life (secondary outcome).
8. Collect and explore people in custody’s experiences of receiving BA.
9. Report the number of adverse events and harms that occur during the trial.

## Method

This a two-arm feasibility randomised controlled trial. The design of this protocol is informed by the Standard Protocol Items: Recommendations for Interventional Studies (SPIRIT 2013) statement: defining standard protocol items for clinical trials [18] (see Supporting Information. Supplementary file. Schedule of enrolment, interventions, and assessments). The Consolidated Standards of Reporting Trials (CONSORT) 2010 statement: extension to randomised pilot and feasibility studies will guide the reporting of findings from the study [19].

### Setting

**Fig 1:**
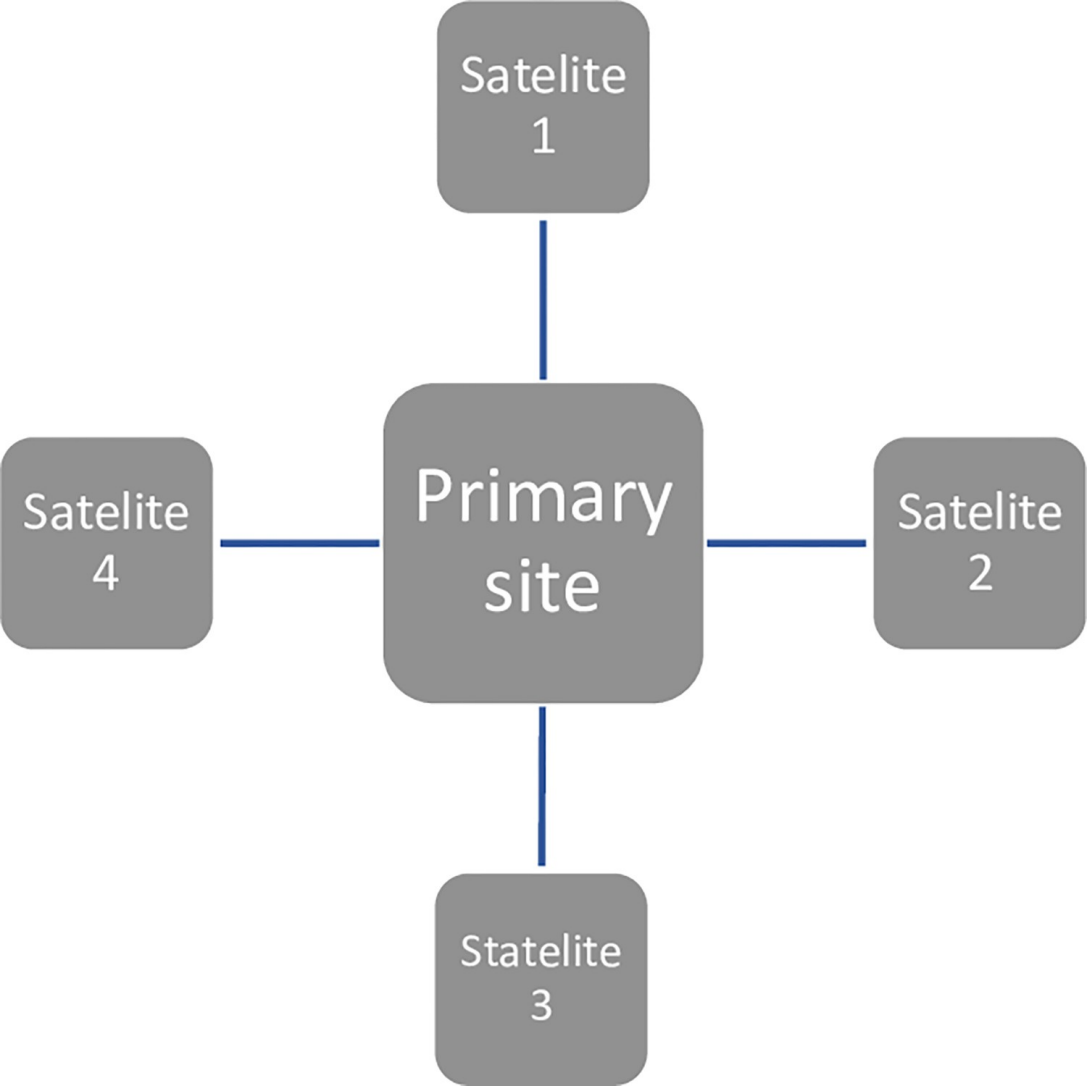
Tele trial cluster.

Our feasibility trial will be implemented at four South Australian prison sites in which primary health care services are provided to people in custody by the Central Adelaide Local Health Network (CALHN) (see Fig 1). A primary site located within the region will cater as the coordinating centre.

### Participants

The study will comprise two groups of participants: custodial health nurses and people in custody with depressive symptoms.

### Selection criteria

#### Inclusion criteria

- Custodial health nurses
  Custodial health nurses must (i) be employed by CALHN, (ii) be actively involved in delivery of direct care to people in custody, (iii) successfully completed the online certificate in BA, and (iv) agreed to participate in the study.
- People in custody
  People in custody will be recruited if they are (i) aged 18 years or above, (ii) receiving a primary health care service by SA health nurses provided by CALHN at any of the four participating prison sites, (iii) experiencing mild to moderate depressive symptoms as demonstrated with a baseline score of 5–14 (mild to moderate depression) [20] on the PHQ-9 scale, (iv) able to understand, read, write, and speak English, and (v) capable of giving informed consent. Prior or current use (including during the trial) of medication or psychotherapy will not be a precluding factor.

### Exclusion criteria

- Custodial health nurses

Custodial health nurses who indicate they will leave their position within the next 12 months.

- People in custody

People in custody will be excluded from the study if they (i) have depressive symptoms and achieve a baseline score of 15+ on the PHQ-9 scale or acute depression. These people will be excluded from the study for their safety, (ii) express suicidal ideation or are at risk of self-harm, suicide, homicide, or present a risk to others. These potential participants will be referred to a general practitioner or mental health professional for support, (iii) have multiple mental health diagnoses, where depression is secondary to another primary mental health diagnosis, (iii) have a Department for Corrections (DCS) Notice of Concern (NOC) and are placed for High-Risk Assessment Team (HRAT) review, (iv) are actively being treated by Mental Health or Forensic Mental Health team, and (v) have a disorder that impedes effective communication (eg, severe sensory impairment) or have trouble communicating effectively in English.

### Sample size

Our target sample size is 60 people in custody and eight custodial health nurses. The sample size estimation is based on the median sample size per arm of 30 recommended for feasibility studies with continuous outcome measures [21], such as levels of depressive symptoms.

### Intervention group

Thirty people in custody will be recruited to the intervention group, who will receive BA plus usual prison health care.

### TAU group

Thirty people in custody allocated to the control group will receive usual prison health care alone. No other specific intervention will be provided to this group.

### Recruitment

a) Custodial health nurses

Initial contact with the custodial health nurses who have completed an online training program in BA will be made via email by one of the researchers (MJ). If the custodial health nurse expresses an interest in participating in the trial, the trial nurse will contact potential participants via email, providing an information sheet about the research and a consent form. A trials nurse will be available to answer participants’ questions via email or phone. The trial nurse will follow up with the potential participant after one week to establish whether the custodial health nurse is willing to participate in the research and return the signed consent form. The custodial health nurse can withdraw from the study at any time.

The trial nurse, employed by South Australia Regional Tele Trials Scheme and not an employee of the South Australia Prison Health Service, will work collaboratively with the staff of the prison health service to identify and recruit potential participants. An information sheet will be provided and discussed with the custodial health nurses that volunteer to participate in the trial after completing the BA training. The custodial health nurses who agree to participate will be asked to review the informed consent form and confirm their agreement to participate in the study by signing it.

b) People in custody

Preliminary identification of the potential people in custody will be done by the custodial health nurses. The custodial health nurses will determine if the people in custody meet the broad eligibility criteria set for the clinical trial. The custodial health nurses will briefly inform the people in custody about the study and offer them a Participant Information Sheet. People in custody consenting to participate in the study and to having their details shared will then be approached by the trial nurse. The trial nurse will provide the people in custody with a detailed study information sheet after assessing if the people in custody meet the inclusion criteria including experiencing depressive symptoms. The trial nurse will offer further explanation or clarification as required and address concerns the people in custody may have. People in custody will be given at least 48 hours to consider their involvement with the study. People in custody that agree to participate in the study will be invited to sign an informed consent form before being enrolled in the study.

At the end of the study, a sub-sample of custodial health nurses (six) and people in custody (fifteen, including those who do, and do not, fully complete the BA sessions) will be invited to participate in individual, semi-structured interviews. The trial nurse will contact the custodial health nurses and the people in custody about the interviews.

### Random treatment allocation and blinding

The trial nurse will collect baseline data from the people in custody who voluntarily agree to participate in the study. The people in custody will then be randomly assigned into either: (a) the BA group, who will receive BA plus usual prison health care, or (b) the Treatment as usual (TAU) group, who will receive the usual prison health care alone.

An external computerised randomisation service will be contracted to generate random allocation once relevant baseline data has been collected. Block randomisation with random permuted block sizes will ensure appropriate treatment allocation concealment and equal sample sizes across groups. The trial nurse will insert the unique participant ID into Sealed Envelope (sealedenvelope.com), an online software application for randomising patients into clinical trials [22]. The online computer application will generate treatment allocation and send an email confirmation of the group allocation to the trial nurse. The allocation sequence is retained by the contracted service and the trial nurse will be unaware of group allocation until receiving confirmation for each participant. The trial nurse will then inform the people in custody and custodial health nurses of treatment allocation. Allocation will be blinded to the data manager/statistician, but it will not be masked to the custodial health nurses and trial nurse who will collect the baseline, post-intervention, and follow-up outcome data.

**Fig 2:**
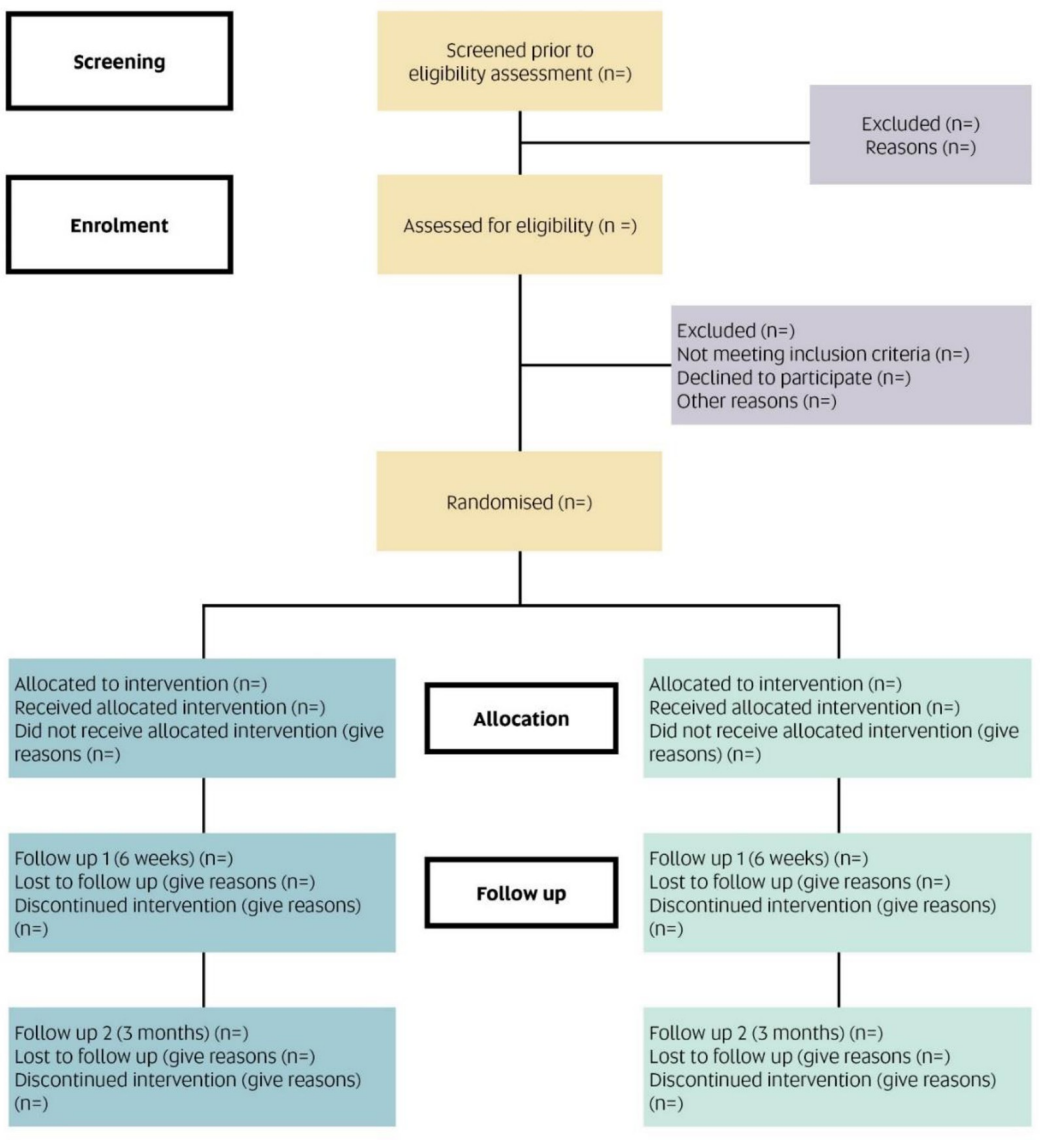
CONSORT flow diagram.

The CONSORT diagram illustrating participant flow is shown below (see Fig 2).

### BA intervention

The BA intervention focuses on increasing engagement in activities than have a sense of pleasure or mastery, while minimising engagement in activities that may be associated with depression. Sessions are structured so the nurse works collaboratively with the person in custody, to identify the relationship between behaviour and mood, and change behaviour that could sustain depressed mood (See Supplementary file. Session guide for custodial nurses). Nurses will deliver the BA intervention in the prison health facility, as per usual practice. In consultation with the nurses at each of the prison sites, a list of possible activities that people in custody can engage in has been developed. This is based on the available resources and level of security in each allocated residential unit.

A recent meta-analysis of RCTs examining individually deliver BA, identified a range in the number of BA sessions conducted from 1 to 24, however most studies adopted 4 to 12 BA sessions [23]. In this trial, we have proposed 12 twice weekly 30-minute sessions of BA, with a minimum of 6 for a participant to be considered as having the BA intervention. This is within the range of commonly accepted practice [23].

### BA training and supervision

The custodial health nurses that join the study will have completed BA training via an academically accredited online “Professional Certificate in BA for Depression” offered by the University of South Australia. The 10-week online training program is fully described in an earlier study by the same authors and consists of five modules including, (a) the evidence base of BA (b) introduction to BA (c) assessment and mood monitoring (d) functional analysis, and (e) activity scheduling [24]. The Professional Certificate has two assessment components. Knowledge of BA is assessed with a series of multiple-choice questions at the end of each module. While competency is assessed through the submission of three videos demonstrating the core aspects of BA, demonstrating the skills the student has learned through the program. These role-played assessments are completed by the student with a work colleague, or another student from the course, as the recipient of BA.

Custodial health nurses that complete the training will deliver BA to participating people in custody with supervision being provided via telehealth by MJ, SD, and SW. Each custodial health nurse will deliver BA to at least five people in custody.

### Intervention fidelity

We will assess the quality of and adherence to the BA treatment protocol via a telehealth supervision and support structure provided by the BA experts. A checklist developed as part of the online BA training will be used to assess quality and adherence in a standardised manner.

### Process evaluation (acceptability and satisfaction)

Individual, semi-structured interviews will be conducted with a purposively sampled subset of custodial health nurses (minimum of 6) and people in custody (minimum of 10). We will explore the participants’ experiences of involvement in the trial. Interviews will be conducted with custodial health nurses to gain insight into their experiences of completing the BA training program and delivering the BA treatment. The custodial health nurses will also be invited to share their experience of the supervision provided by the BA experts via the telehealth platform. The interviews with people in custody will focus on their experiences of, and perceived impact of, engaging in the BA treatment. General topic areas (for people in custody and custodial health nurses) will include what worked; what did not work; what could be done differently; what could be improved; and what were the facilitators and barriers to engaging with BA.

The interviews will be conducted by the trial nurse or an academic team member (in-person or by phone), audio-recorded (where feasible), and are anticipated to last no more than 30 minutes. Data collection will cease once data saturation is achieved; that is, the information becomes too repetitive and no new insights are added by the interviews [25]. An interview topic guide will be developed for each group of interviewees.

### Primary clinical outcome and measure

The primary clinical outcome will be a change in depressive symptoms as assessed by the trial nurse using the Primary Health Questionnaire Nine (PHQ-9) scale [26,27]. The PHQ 9 is a 4-point Likert-type scale (0, absent; 1, mild; 2, moderate; 3, severe) with nine items that correspond to the DSM-IV Diagnostic Criterion A symptoms for major depressive disorder [28]. A meta-analysis rated the PHQ-9 scale as being reliable with a sensitivity of 88% and a specificity of 78% at a cut-off of 10 or above [29]. The primary clinical outcome measure will be implemented at three-time points (a) baseline (0 weeks), (b) 6 weeks post-intervention, and (c) 3 months post-intervention.

### Secondary clinical outcome and measure

The secondary clinical outcome will be the level of health-related quality of life as measured by the 36-Item Short Form Health Survey (SF 36) [30] The SF 36 measures physical functioning, role physical, bodily pain, general health, vitality, social functioning, role emotion, and mental health [30]. In this study, we will administer the SF 36 questionnaire at three time points: (a) at baseline (0 weeks), (b) 6 weeks post-intervention, and (c) 3 months post-intervention.

### Other data to be collected

#### Sociodemographic questionnaire

Demographic information about gender, date of birth, marital status, educational level, and type of prison pathway person in custody is located will be collected by the trial nurse at the assessment and recruitment stage.

#### Contextual factors

We will collect information about each participating tele-trial site at baseline and 6 weeks post-intervention. The information will be important in understanding the implementation process. These factors (including organisational changes, staffing changes, etc), will be collected by the trial nurse through conversations with the custodial health nurses and the nurse unit managers at the four participating prisons.

### Data collection and management

The data manager will facilitate the data collection process. With prior written consent, participant demographic information will be collected through the tele-trial sites. With permission from people in custody, outcome data will also be collected and analysed from those that discontinue the study or deviate from the intervention protocol.

The interviews with the people in custody and custodial health nurses will be audio-recorded (where feasible) with permission from the interviewees. The audio recordings will be transcribed verbatim by independent transcribers contracted by the University of South Australia. The transcription service signed confidentiality and data security agreements with the University. Transcripts will be returned to the originators as part of transcript review and validation [31]. Transcripts will be de-identified prior to analysis.

All collect data will be securely stored in lockable cabinets or access-restricted computers and servers with the individually identifiable data kept separately from other research data to maintain confidentiality and anonymity. Following the National Health and Medical Research Council (NHMRC) guidelines for clinical trials, data will be retained for 15 years and then securely destroyed. Data will be managed following the National Statement on Ethical Conduct in Human Research [32] and the University of South Australia Research Data Management Procedures.

### Qualitative data analysis

The qualitative data from interviews will be imported into NVivo Pro software version 12. The Thematic analysis, as described by Braun and Clark [33], will then be undertaken. This will encompass the processes of immersion in the data set, coding to generate preliminary codes, and the development and revision of tentative themes. This will be followed by a description of the themes and sub-themes. The interview transcripts will be examined thematically across the entire data set as well as based on each interview. Codes and themes will be validated by an independent researcher at key points in the analysis.

### Quantitative data analysis

Quantitative data will be collected using Qualtrics online survey platform. We will describe the demographic characteristics of the custodial health nurses and people in custody using Microsoft Excel software [34] or IBM SPSS Statistics software version 29 [35]. The descriptive data will be presented and reported using mean, standard deviation, median, and range or counts and percentages.

### Statistical analysis

The primary analysis is intended to determine whether conducting a subsequent fully powered RCT is feasible. Therefore, the analyses will be descriptive by exploring all feasibility outcomes and will include measures of uncertainty, such as 95% CIs. As feasibility trials are not designed to establish efficacy, we will estimate the variance of outcome measures and calculate the effect size differences (with 95% CIs) on outcome measures from baseline to both follow-up points (on an intention-to-treat basis) in both groups. Where appropriate, the generalised estimating equation (GEE) will be employed to analyse the preliminary effects of BA across two time points (baseline and 6 weeks). The GEE analysis will account for intra-correlated repeated outcome data and accommodate data missing at random.

### Dissemination

We will report the findings using the Consolidated Standards of Reporting Trials (CONSORT) framework for reporting randomised pilot and feasibility trials [36]. We intend to publish the findings in peer-reviewed journals. Authorship guidelines will be consistent with those proposed by the International Committee of Medical Journal Editors policy [37]. Findings will also be presented at relevant seminars and professional conferences. Additionally, the participating prison sites will be provided with a summary of the findings for distribution to participants and relevant stakeholders.

### Ethical considerations

This study will be informed by the principles of Good Clinical Practice (GCP) enshrined in the “Declaration of Helsinki”, the Australian Code for the Responsible Conduct of Research, and the Australian Clinical Trial Handbook [38–40]. This study has been approved by Central Adelaide Local Health Network Human Research Ethics Committee (REF. NO. 2023/HRE00097 dated 28 June 2023) and the University of South Australia Human Research Ethics Committee (REF. NO. 205681 dated 3 August 2023). Public access to the study protocol has been ensured via the publication and registration of the trial protocol in the Australian New Zealand Clinical Trials Registry (ANZCTR registration ACTRN12623000346673p) [41]. We will follow the policy and guidelines of the Australian Code for the Responsible Conduct of Research [40], and the Australian Clinical Trial Handbook [39] on sharing and releasing data. The final trial dataset will be deidentified and not include any participant identifiers.

BA will be delivered by the custodial health nurses who will be responsible for recognising, responding, and reporting any distress arising from the intervention. The custodial health nurses will receive ongoing clinical supervision from BA experts who will have adequate professional indemnity insurance. After a person in custody has completed each session of BA, the custodial health nurses will ask them about their emotional well-being and if they have experienced any distress. If distress is experienced, the people in custody will have access to ongoing support through their existing support team and will be aware of how to contact them. Any modifications to the protocol will be advised to the respective Ethics Committee and the Australian New Zealand Clinical Trials Registry (ANZCTR), accordingly.

### Patient and public involvement

An advisory committee will be established to develop, refine, and review the study recruitment materials. This will comprise prison advocacy groups from the four sites. We will ask the prison advocates for advice on interpreting the interviews conducted with people in custody and on how best to share the findings with other patient-public involvement groups who support people in custody.

### Safety considerations

As noted, to our knowledge BA has not been conducted in SA prisons before. Thus, our safety considerations have been informed by other studies conducting similar interventions in community dwellings. An RCT study investigating the cost-effectiveness of BA compared with CBT for adults with depression reported that depression-related, but not treatment-related, serious adverse events occurred in three participants in the BA group and eight participants in the CBT group [16]. The workers who delivered BA in the study were not specialist mental health workers, but university graduates who completed a five-day training program in BA.

To mitigate the risk of adverse effects we will put in place several procedures. We will ensure that custodial health nurses engage the people in custody at each assessment or the start of each BA session to check how they are feeling and if they experienced any unexpected effects of the intervention.

Custodial health nurses may find it distressing to work with people in custody with depression. Opportunities will be provided for the custodial health nurses to debrief with the BA experts via the telehealth supervision and support structure. In addition, all custodial health nurses will be invited to attend monthly supervision sessions with the BA experts.

We will report all adverse events and serious adverse events to the steering group and relevant ethics committees. If the steering group considered that adverse events were a result of the intervention, we will suspend the trial. Adverse events will be analysed and, where related to delivery mechanisms, will inform modifications to future trials.

### Steering group

We will establish a Tele-trial Steering Group (TSG) comprising the research team, two prison advocates, and two independent members to provide safety oversight on the tele-trial processes including trial management and monitoring. They will independently review outcome data and any adverse events relating to the trial. The TSG will meet at least twice per year for the duration of the trial.

### Anticipated timeline

The delivery of BA is expected to commence in January 2024. Recruitment of people in custody will occur from November until December 2023 to be followed by the collection of baseline data. Follow-up will occur at 6 weeks and again at 3 months post-intervention. The trial will finish in June 2024.

## Discussion

As people in custody are at risk of developing depression, identifying, and testing appropriate intervention options is important. A systematic interview identified several RCTs, conducted in prisons, to improve the mental health of people in custody [42]. Individual and group CBT have both demonstrated improvements in mental health of male prisoners, with group CBT showing more improved outcomes [42]. Dialectical behaviour therapy was shown to improve mental health outcomes for people in custody with impulsive behaviour issues [42]. However, access to psychological interventions in prison settings can be challenging. In rural areas of Australia, access to psychological services can be challenging for several reasons [43]; custodial settings in these areas may face the same issues, including a lack of access to qualified psychological staff. Therefore, it is important to consider interventions that can be delivered by other health and social care professionals. As BA has shown to be as effective as CBT in non-custodial settings and can be delivered by other professional groups including nurses [16], it is a worthwhile consideration.

A potential challenge to delivering BA in a prison setting is the restricted environment. Participants do not have the range of options available to people living in non-custodial settings. This makes feasibility an important consideration. However, BA could encourage people in custody to consider what available activities they find enjoyable. As noted by Magaletta et al. [44], scheduling enjoyable activity in a prison environment could have psychological benefit and could help “structure inmate accountability strategies and practices” (p.119). With this in mind, Magaletta et al. [44], were able to develop a daily activities list for people in custody that contained 227 items. Meeks et al. [45] were able to implement BA in a custodial setting with four older prisoners. A therapist, external to the prison, worked with prison activity staff to deliver the BA intervention to medically frail prisoners. While the authors considered the case study demonstrated feasibility for BA in prison settings, they noted several factors that could impede its implementation. This included under-resourcing, a lack of privacy for therapy sessions, and a potential reluctance of people in custody to engage with strangers about their mental health. These studies indicate the potential for BA to be applied in restricted environments. Our approach to custodial nurses delivering the intervention overcomes some of the identified barriers, including accessing specialist mental health workers, a level of privacy when delivering the intervention, and having a level of trust to disclose mental health concerns.

This trial has several innovations: incorporation of a real-time fidelity and supervision structure, blended quantitative and qualitative design elements, and task-shifting from psychologists and mental health workers to primary care custodial health nurses. Cuijpers et al. [23], noted that many of the studies included in their meta-analysis of BA, did not include information regarding harms or adverse effects of BA exposure. In this study, we will collect information regarding all harms that occur to the participants (custodial nurses and people in custody), and report these to the trial steering group. The trial steering group will consider whether these harms are attributable to the intervention. Further, all harms will be reported in publications emanating from the study.

A key advantage of using BA with the study population is that it is easy and relatively inexpensive to implement by workers who are not specialist mental health workers [46–48]. This may provide opportunities for scaling up BA across prison sites in Australia and overseas to support people in custody living with depression.

### Strengths

The proposed study aims to develop and test the feasibility and acceptability of BA in treating depression in people in custody. The study has potential to generate wide economic and social benefits through scaling-up beyond psychologists and mental health workers to an environment in which people are at higher risk of depression. The simplicity of the model if feasible may allow implementation of BA in other prison environments in Australia and other countries.

### Limitations

The trial may experience challenges reaching the target sample size within the anticipated timeframe, leading to possible project delays. While the trial may provide some preliminary insight into differences in clinical outcomes, the study will not be powered to detect statistically significant differences. Due to potential restrictions and challenges (e.g., lockdowns, staff shortages, limited access to therapy spaces) of people in custody accessing and attending BA sessions, the timeline for this trial will be flexible. For similar reasons, a flexible and pragmatic approach to data collection will be adopted.

## Conclusion

BA is an effective treatment for depression, however, we do not yet understand its potential to support people in custody with depression when delivered by custodial health nurses. An important first step is to understand if BA is acceptable and feasible. Our feasibility trial will be undertaken in partnership with the Central Adelaide Local Health Network. As far as we are aware, this trial will be the first to establish the acceptability and feasibility of BA for this population. Our data regarding the completion of the outcome measures and the interview data will help us understand the feasibility and acceptability of the treatment and the outcome measures. The feasibility data relating to participants who withdraw from our study will help us to calculate the number of participants we need to recruit. If feasible and acceptable, the results from this proposed study will help us to design and conduct a definitive randomised controlled trial.

## Supporting information

S1 Checklist SPIRIT 2013 checklist: Recommended items to address in a clinical trial protocol and related documents*. (DOC)

S1 File (DOCX)

S2 File Session guide for custodial nurses. (DOCX)
