# Supplementary material for: Behavioural activation for people in custody with depression: A protocol for a feasibility randomised controlled study
Source: PLoS One. 2024 Jun 13;19(6):e0304767. doi: 10.1371/journal.pone.0304767 (PMC11175500; doi:10.1371/journal.pone.0304767)
Supplement: S1 File — (DOCX) [file pone.0304767.s002.docx]

**Full title: Co-delivery of tele trial Behavioural Activation in people in custody with depression: a Randomised Controlled Trial feasibility protocol**

**Short title: Tele trial Behavioural Activation for people in custody with depression**

**Research Team**

Associate Professor Martin Jones, Project Director University of South Australia Department of Rural Health Mount Barker, South Australia

Steven Hutchinson, Nurse Consultant – Training & Practice Development, South Australian Prison Health Service, SA Health Central Adelaide Local Health Network, South Australia

Dr Pascal Dettwiller, Liaison Officer, SA Regional Clinical Trials Co-ordinating Centre, Office for Research, Department for Health, and Wellbeing, South Australia

Dr Thomas Turnbull, Medical Director, South Australian Prison Health Service, SA Health Central Adelaide Local Health Network, South Australia

Lucy Howard, Nurse Unit Manager, South Australian Prison Health Service, SA Health Central Adelaide Local Health Network, South Australia

Jo Andrews, Nurse Unit Manager, South Australian Prison Health Service, SA Health Central Adelaide Local Health Network, South Australia

Dr Kuan Liung Tan, Program Director: Bachelor of Community Health, Bachelor of Health Sciences UO, UniSA Clinical and Health Sciences, University of South Australia

Shaun Dennis, Senior Mental Health Project Officer, Rural and Remote Mental Health Service, Barossa Hills Fleurieu Local Health Network, South Australia

Professor Dan Bressington, Professor of Nursing, Charles Darwin University, Northern Territory

Professor Richard Gray, Theme Lead, Healthy People Families and Communities La Trobe University, Victoria

Dr Shyamsundar Muthuramalingam, Consumer Engagement, South Australian Prison Health Service, SA Health Central Adelaide Local Health Network, South Australia

Sandra Walsh, Research Assistant, University of South Australia Department of Rural Health, Whyalla, South Australia

Kuda Muyambi, Research Assistant, University of South Australia Department of Rural Health Mount Barker, South Australia

Professor Vincent L Versace, Director, Deakin Rural Health, Deakin University, Warnambool, Victoria

**Project sponsor**: University of South Australia

**Abstract**

People in custody are at high risk of developing depression. Accessing psychological treatments such as Cognitive Behavioural Therapy (CBT) in a prison setting is a particular challenge in part due to difficulties in accessing specialist mental health workers. An alternative treatment, Behavioural Activation (BA), is just as effective as CBT for depression in adults who are not in prison settings. BA works by teaching people the skills to notice fluctuations in their mood and notice behaviours they were engaging in. The second step is to help people schedule activity which results in an increase in mood. The application of BA may increase access to effective treatment for people in custody with depression. However, we lack trial data concerning its acceptability and feasibility. The aim of the study is to examine the feasibility and acceptability of telehealth BA plus usual prison health care. Findings from the study may provide opportunities for scaling up BA to support people in custody living with depression.

**Method**

A sample of 60 people in custody aged over 18 years or older with presenting symptoms of depression as assessed using the PHQ 9 scale (with a score of 5-14 mild to moderate depression) will participate in the trial. Recruited people in custody will be randomised to receive BA plus treatment as usual (TAU) or TAU for depression provided by custodial health nurses. Eight custodial health nurses will be recruited from four rural prisons in South Australia. The custodial health nurses will be prepared to deliver Behavioural Activation to people in custody at the selected prisons. The BA intervention will involve preparing people in custody to monitor their mood, identify triggers that can impact mood, identify activities that may enhance mood, and enable each participating person in custody to formulate an activity schedule. BA will involve twelve conversations with people in custody held twice weekly over six weeks. Changes in depressive symptoms and health-related quality of life will be assessed at three-time points: (a) at baseline, (b) 6 weeks, and (c) 3 months post-intervention. We will use descriptive statistics to report changes over time, but not make any judgement. In addition, we will interview custodial health nurses and people in custody to understand further BA acceptability and feasibility in prison settings.

**Discussion**

The findings will inform the design of a full-scale randomised controlled trial to test the efficacy of co-delivered tele-trial BA for people in custody with depression.

**Trial registration**

Australian New Zealand Clinical Trials Registry (ACTRN12623000346673p)

**BACKGROUND**

People in custody are at an increased risk of developing mental health problems, particularly depression (1-3). In a systematic review of 109 studies involving 33, 588 people in custody conducted in 24 countries during the period 1966 to 2010, the authors reported a pooled prevalence of major depression of 10.2% (95% CI 8.8–11.7) in male and 14.1% (95% CI 10.2–18.1) in female people in custody (4). Depression is often underdiagnosed and under-treated in prison settings (1). Treating depression in people in custody can have several economic and social benefits. For instance, treating depression in people in custody reduces the risks of suicide (5) and self-harm (6) whilst in custody and premature mortality (7), recidivism (8), and violence and victimisation (1) on release from prison.

The recommended treatments for depression include antidepressant medication and psychotherapy used singly or in combination (9). However, the use of antidepressants in custodial settings is problematic such as problems with adherence to antidepressants or misuse and adverse effects (10). CBT is the recommended treatment for depression in adults (9). CBT is effective in treating depression in people in custody (11, 12). However, accessing CBT in prison health care environments is challenging. Typically, you need to be a specialist mental health worker (Psychologist, Occupational Therapist, or Mental Health Nurse) to deliver BA. Accessing these types of specialist mental health workers in prisons is challenging. An alternative psychotherapy to CBT is Behavioural Activation (BA). A comparison of the effectiveness of CBT and BA in the treatment of depression indicated that both psychotherapies are equally effective (13). BA is a derivative of CBT but works as a standalone psychotherapy. BA works by teaching people the skills to notice fluctuations in their mood and notice behaviours they were engaging in. The second step is to help people schedule activity which results in an increase in mood. A strength of BA is you do not need to be a specialist mental health worker and training health care workers to practice the approach involves a relatively short – five days – in comparison to CBT – 18 months. The theoretical underpinning of BA is that people improve their mood if they routinely engaged in pleasurable events and minimised avoidance (14, 15).

We do not yet understand if BA is efficacious in treating depression in adult people in custody when delivered by non-specialist mental health workers. This clinical trial will investigate the feasibility of preparing custodial health care nurses to deliver BA to support people in custody with depression. If the clinical trial demonstrates that BA delivered by custodial health nurses is efficacious in treating depression in people in custody, this may help to increase access to evidence-based psychological treatment for people in custody living with depression.

**AIMS**

We will examine the feasibility and acceptability of:

1. Telehealth BA plus usual SA prison health care (intervention) compared to usual SA prison health care alone (treatment as usual (TAU)) in the treatment and management of depression in people in custody who have been diagnosed with a mood disorder.
2. Telehealth BA plus usual SA prison health care (intervention) compared to usual SA prison health care alone (TAU) in improving the quality of life of people in custody who are diagnosed with clinically meaningful depressive symptoms.

**Objectives**

**Custodial health nurses**

1. Establish custodial health nurses' fidelity to BA treatment.
2. Explore custodial health nurses' experiences of delivering BA treatment to people in custody supported by telehealth.
3. Explore the custodial health nurses' experiences receiving co-delivered tele-trial BA training and supervision.

**People in custody**

1. Establish the number of people in custody accessing the South Australian Prison Health Service (SAPHS) who report experiencing clinically meaningful depressive symptoms.
2. Determine the number of people in custody with clinically meaningful depressive symptoms who are approached and agree to participate in the trial.
3. Determine the proportion of eligible people in custody who have agreed to participate and agree to be randomised.
4. Determine the proportion of people in custody with clinically meaningful depressive symptoms that participate in the trial who complete baseline measures.
5. Determine the proportion of people in custody who complete a co-delivered telehealth BA treatment (attending for a minimum of six sessions over six weeks)
6. Determine the proportion of people in custody who complete outcome measures at six weeks and three-month follow-up.
7. Calculate the preliminary efficacy of BA on depressive symptoms (primary outcome) and improving health-related quality of life (secondary outcome).
8. Explore people in custody's experiences of receiving a co-delivered telehealth BA.
9. Report the number of adverse events and harms that occur during the trial.

**METHOD**

**Design**

We will evaluate the feasibility and acceptability of BA plus usual SA prison health care in reducing depressive symptoms in people in custody compared to SA prison health care alone (TAU). The design of this protocol is informed by the Standard Protocol Items: Recommendations for Interventional Studies (SPIRIT 2013) statement: defining standard protocol items for clinical trials (16). The Consolidated Standards of Reporting Trials (CONSORT) guidelines will guide reporting of the trial. Consolidated criteria for Reporting Qualitative research (COREQ) guidelines will be followed for the reporting of findings from interviews or focus groups (17, 18).

**Setting**

Our feasibility trial will be implemented across a cluster of four South Australian prison sites (Murray Bridge, Port Augusta, Port Lincoln, and Cadell) in which primary health care services are provided by Central Adelaide Local Health Network (through Prison Health Services) to people in custody. (Figure 1).

Figure 1 Tele trial cluster

**Participants**

***Inclusion criteria***

The study will comprise two groups of participants: Custodial health nurses and people in custody with depressive symptoms.

1. Custodial health nurses:
2. employed by CALHN
3. Complete the Professional Certificate in Behavioural Activation
4. agree to participate in the study.
5. People in custody that are:
6. Aged 18 years or above.
7. Receiving a primary health care service by SA health nurses provided by CALHN at any of the four participating prison sites.
8. Experiencing mild to moderate depressive symptoms as demonstrated with a baseline score of 5-14 (mild to moderate depression) (19) on the PHQ-9 scale.
9. Understand, read, write, and speak English.
10. Provide informed consent.

Prior or current use of medication or psychotherapy will not be a precluding factor.

***Exclusion criteria***

- People in custody with depressive symptoms who achieve a baseline score of 15+ on the PHQ-9 scale will be excluded from the study for their safety.
- People in custody who express suicidal ideation or present a risk to themselves, or others will be excluded. These potential participants who are excluded will be referred to a general practitioner or mental health professional for support. People in custody will be excluded if they display acute depression and need to be seen by a psychiatrist.
- People in custody with multiple mental health diagnoses.
- A Department for Corrections (DCS) Notice of Concern (NOC) and placed for High-Risk Assessment Team (HRAT) review.
- People in custody who are at risk of self-harm, suicide, or homicide.
- People in custody who are actively being treated by Mental Health or Forensic Mental Health team.

**Sample size**

Our target sample size is 60 people in custody and eight (8) custodial health nurses. We will determine the sample size estimation based on the median sample size per arm of 30 recommended for feasibility studies with continuous outcome measures such as levels of depressive symptoms (20).

**Recruitment**

1. Custodial health nurses

Initial contact with the custodial health nurses who complete the BA training will be made via email by PI MJ (See Attachment VI). If the custodial health nurse expresses an interest in participating in the trial, the tele-trial nurse will contact potential participants via email, providing an information sheet about the research and a consent form. The tele-trial nurse will be available to answer participant questions via email or phone. The tele-trial nurse will follow up with the potential participant after one week to establish whether the custodial health nurse is willing to participate in the research and return the signed consent form. The custodial health nurse can withdraw from the study at any time.

The tele-trial nurse is employed by South Australia Regional Tele Trials Scheme and is not an employee of the South Australia Prison Health Service. The role of the tele-trial nurse is to support the Principal Investigator to conduct a clinical trial geographically remote from the Principal Investigator’s primary site. They will work collaboratively with the site-specific staff of the SAPHS to identify and recruit potential participants. In addition, the tele-trial nurse will be responsible for data collection including key outcome measures and interviews.

1. People in custody

Preliminary identification of the potential people in custody will be done by the custodial health nurses. The custodial health nurses will screen if the people in custody meet the eligibility criteria set for the clinical trial. The nurses will briefly inform the people in custody about the study and offer them an information sheet. Consenting people in custody will be approached by the research tele-trial nurse. The research tele-trial nurse will then assess if the people in custody meet the inclusion criteria including experiencing depressive symptoms before handing out a detailed study information sheet. The research tele-trial nurse will offer further explanation or clarification as required and address concerns the people in custody may have. The people in custody will be given at least 48 hours to consider their involvement with the study.

At the end of the study, we will invite a sub-sample of custodial health nurses and people in custody to participate in individual, semi-structured interviews. Four to six custodial health nurses and 10 to 20 people in custody will be interviewed. The research tele-trial nurse will contact the custodial health nurses and the people in custody about the interviews.

**Random treatment allocation and blinding**

The research tele-trial nurse will collect baseline data from the people in custody who voluntarily agree to participate in the study. The people in custody will then be randomly assigned into either: 1) the Behavioural Activation group, which will receive BA plus usual prison health care, or 2) the TAU group, which will receive the usual prison health care alone.

An external computerised randomisation service will be contracted to generate random allocation. Block randomisation with random permuted block sizes will ensure appropriate allocation concealment and equal sample sizes across groups. Following baseline data collection from consenting people in custody, the research trial nurse will insert the unique participant ID into the online randomisation service (sealedenvelope.com) and receive an email confirmation of their group allocation. The allocation sequence is retained by the contracted service and the research trial nurse will be unaware of group allocation until receiving confirmation for each participant. The tele-trial research nurse will then inform the people in custody and the custodial health nurses of treatment allocation. Allocation will be blinded to the data manager/statistician, but it will not be masked to the custodial health nurses and research trial nurse who will collect the baseline, post-intervention, and follow-up outcome data.

**Intervention**

**BA tele-trial intervention group**

We will recruit approximately 30 people in custody in the intervention group who will receive BA plus usual prison health care (20). The intervention will be delivered by the custodial health nurses who will receive ongoing clinical supervision from an expert in BA via a telehealth video link. The people in custody will be offered twelve sessions of BA, which will be delivered twice weekly, in 30-minute sessions, for six weeks. Specific BA techniques that will be applied include (a) identification of depressed behaviours (b) analysis of the triggers and consequences of depressed behaviours (c) monitoring of activities (d) development of alternative goal-orientated behaviours (e) scheduling of activities, and (f) the development of alternative behavioural responses to rumination.

**TAU group**

People in custody allocated to the control group will receive the usual prison health care alone. No other specific intervention will be provided to this group. Across sites, TAU will be composed of 30 participants (20).

The CONSORT diagram illustrating participant flow is shown below (Figure I).


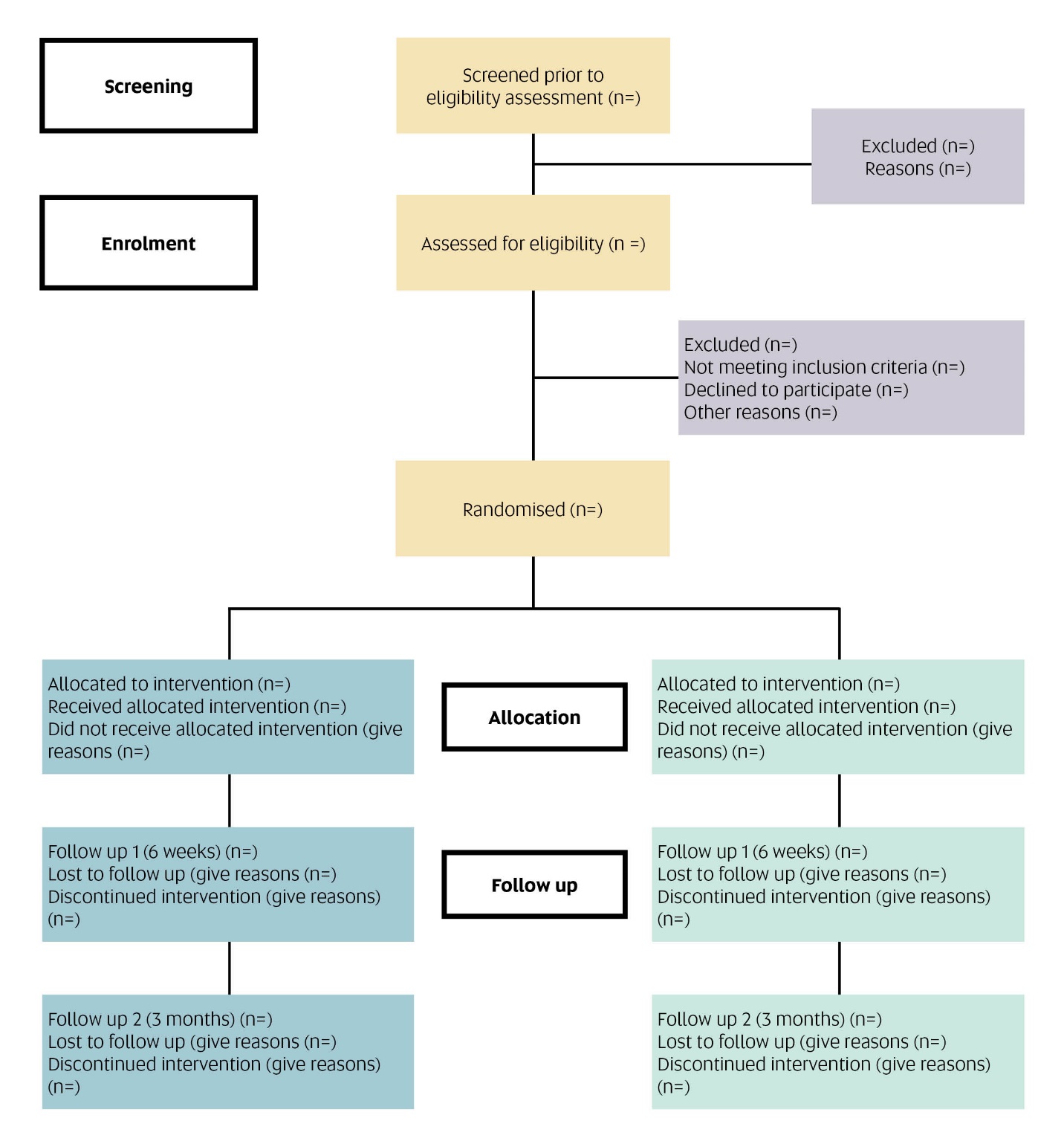


**BA training**

The training in BA techniques is an academically accredited online “Professional Certificate in BA for Depression” program offered by the University of South Australia. The 10-week online training program consists of five modules: (a) the evidence base of BA (b) introduction to BA (c) assessment and mood monitoring (d) functional analysis, and (e) activity scheduling. BA experts, MJ, SD, and SW facilitate the training.

Custodial health nurses who have completed the Professional Certificate are expected to have achieved the following learning outcomes: (a) gained a sound understanding of the BA intervention (b) attained the relevant core BA skills, and (c) demonstrated competency in BA delivery.

Custodial health nurses who join the trial have completed the training and will deliver BA to participating people in custody, with supervision being provided via telehealth by MJ, SD, and SW. Each custodial health nurse will deliver BA to at least five people in custody for the duration of the trial.

**Intervention fidelity**

We will assess the quality of and adherence to the BA treatment protocol via the telehealth supervision and support structure.

**Evaluation**

**Feasibility**

The study feasibility outcomes will be evaluated as follows:

- Determine the proportion of eligible people in custody with depressive symptoms who are approached and agree to participate in the trial.
- Determine the proportion of eligible people in custody who have agreed to participate and agree to be randomised.
- Determine the proportion of people in custody with depressive symptoms that complete baseline measures.
- Determine the proportion of people in custody who complete a co-delivered Tele-trial Behavioural Activation treatment (attending for a minimum of six sessions over six weeks).
- Determine the proportion of people in custody who complete outcome measures at six weeks and three-month follow-up.
- Establish SAPHS nurses’ fidelity to Behavioural Activation treatment.
- Calculate the preliminary efficacy of Behavioural Activation on the primary clinical outcome measure.
- Explore SAPHS nurses’ experiences of delivering a co-delivered tele-trial Behavioural Activation treatment to the people in custody.
- Explore SAPHS nurses’ experiences receiving co-delivered tele-trial Behavioural Activation training and supervision.
- Determine the proportion of people in custody with depressive symptoms that participate in the tele-trial improve their health-related quality of life outcomes (clinical outcome measure).
- The number of adverse events and harms that occur during the trial.

**Process evaluation (acceptability and satisfaction)**

Individual, semi-structured interviews will be conducted with a purposively sampled subset of custodial health nurses (minimum of 6) and people in custody with depressive symptoms (minimum of 10) to explore their experiences of involvement in the trial. Interviews will be conducted with custodial health nurses to gain insight into their experiences of completing the BA training program and delivering the BA treatment. The custodial health nurses will also be invited to share their experience of the supervision provided by the BA experts via a telehealth platform.

The interviews with people in custody with depressive symptoms will focus on their experiences of, and perceived impact of, engaging in the BA treatment. General topic areas (for people in custody and custodial health nurses) will include what worked; what did not work; what could be done differently; what could be improved; and what were the facilitators and barriers to engaging with BA.

The interviews will be conducted by the research tele trail nurse, (in-person or by phone), audio-recorded (where feasible), and are anticipated to last no more than 30 minutes. Data collection will cease once the information becomes too repetitive and no new insights are added by the interviews. An interview topic guide will be developed for each group of interviewees.

**Preliminary intervention effects (primary and secondary outcomes)**

**Clinical outcome measure- depression**

The first clinical outcome will be a change in depressive symptoms as assessed by the tele-trial research nurse using the PHQ-9 scale.

*Primary Health Questionnaire Nine (PHQ 9)*. Depressive symptoms will be measured with the Primary Health Questionnaire Nine (PHQ 9) (21, 22). The PHQ 9 is a 4-point Likert-type scale (0, absent; 1, mild; 2, moderate; 3, severe) with nine items that correspond to the DSM-IV Diagnostic Criterion A symptoms for major depressive disorder (23). A meta-analysis rated the PHQ-9 scale as being reliable with a sensitivity of 88% and a specificity of 78% at a cut-off of 10 or above (24).

The clinical outcome measure will be implemented at three-time points (a) at baseline (0 weeks), (b) at 6 weeks post-intervention, and (c) at 3 months post-intervention.

**Clinical outcome measure- quality of life**

The second clinical outcome will be the level of health-related quality of life as measured by the 36-Item Short Form Health Survey (SF 36) (25) The SF 36 measures physical functioning, role physical, bodily pain, general health, vitality, social functioning, role emotion, and mental health (25). In this study, we will administer the SF 36 questionnaire at three-time points: (a) at baseline (0 weeks), immediately after postintervention (6 weeks), and (c) at three months post-intervention.

Completion of eight (8) out of 10 sessions will be deemed to constitute treatment engagement and completion. Acceptability of the treatment will be determined from perceptions of satisfaction with and benefits from the BA treatment obtained through interviews with people in custody.

Other data to be collected

*Sociodemographic Questionnaire*

Demographic information about gender, date of birth, marital status, educational level and type of prison pathway the person in custody is located will be collected by the tele-trial research nurse at the assessment and recruitment stage (Attachment XVII).

*Contextual factors*

We will collect information about each participating tele-trial site at baseline and 6 weeks post-intervention. The information will be important in understanding the implementation process. These factors (including organisational changes, staffing changes, etc), will be collected by the tele-trial research nurse through conversations with the custodial health nurses and the nurse unit managers at the four participating prisons.

**Data collection and management**

The data manager will facilitate the data collection process. With prior written consent, participant demographic information will be collected through the tele-trial sites. For the primary outcome (depressive symptoms), the data will be collected at (a) baseline, (b) 6 weeks, and (c) 3 months post-intervention. The secondary outcome data (health-related quality of life outcomes) will be collected at a) baseline, (b) 6 weeks, and (c) 3 months post-intervention. With permission from people in custody, outcome data will also be collected and analysed from those that discontinue the study or deviate from the intervention protocol.

The interviews with the people in custody and the custodial health nurses will be audio-recorded (where feasible) with permission from the interviewees. The audio recordings will be transcribed verbatim by independent transcribers that are contracted to the University of South Australia. The transcription service signed confidentiality and data security agreements with the university. Transcriptions will be de-identified by the research team before data analysis to ensure anonymity.

The study will collect individually identifiable data (e.g., Consent forms, audio-recordings), non-identifiable data (survey data), and re-identifiable data from which identifiers would have been removed. These types of data will be securely stored with the individually identifiable data kept separately from other research data to maintain confidentiality and anonymity. For example, the audio recordings and corresponding transcripts will be assigned codes that will be kept separately.

Should the need arise to re-identify data, CI KT or PI MJ will be responsible.

All electronic data will be stored securely on the university’s server and files will be password protected. Only the relevant members of the research team will have access to these files. All paper copy data will be kept in lockable drawers at the Whyalla campus of the University of South Australia. Data will be managed following the National Statement on Ethical Conduct in Human Research (26) and the University of South Australia Research Data Management Procedures. De-identified data person in “custody data” and deidentified location data will be made available upon written request, to enable trial checking and reuse.

All the data we collect will be de-identified. Participants' identities will remain confidential. The subject identification log will be kept separately. Chief Investigator (CI) KT and PI MJ will be responsible for de-identifying the data. This will be done by reviewing and removing direct identifiers. Each participant will then be allocated a study number/code. The participant identification code will be kept separately. CIs involved in data analysis and reporting will only have access to the de-identified data; only CI KT and PI MJ will have access to the identification code. Data will be stored on a secure data storage and management system (Qualtrics), hosted by the University of South Australia which will be the institution that owns the data. There are no plans to transfer the data to other sites.

**Data management post-project completion**

After completion of the trial, the data will be continued to be stored on Qualtrics. Only the PI MJ and CI KT will have access to the data after the trial. Following the National Health and Medical Research Council (NHMRC) guidelines for clinical trials, data will be retained for 15 years and then securely destroyed. Files will be destroyed according to the University of South Australia Policy Ownership and Retention of Data (Policy No: RES-17.0)

**Data analysis**

**Qualitative data analysis**

The qualitative data from interviews will be analysed using thematic analysis (27). The planned data analysis will encompass the processes of immersion in the data set, coding to generate preliminary codes, the development of tentative themes, and reflection upon and revision of themes. This will be followed by a description of the themes and sub-themes. The interview transcripts will be examined thematically across the entire data set as well as based on each interview. Codes and themes will be validated by an independent researcher at key points in the analysis. NVivo Pro software version 12 or Qualtrics online survey platform will be employed to facilitate coding (28).

**Quantitative data analysis**

We will describe the demographic characteristics of the custodial health nurses and the people in custody using Microsoft Excel software (29) or the IBM SPSS Statistics software version 29 (30). The descriptive data will be presented and reported using mean, standard deviation, median, and range or counts and percentages.

**Statistical analyses**

All data will be entered and analysed descriptively. The primary analysis is intended to determine whether conducting a subsequent fully powered RCT is feasible. Therefore, the analyses will be descriptive by exploring all feasibility outcomes and will include measures of uncertainty, such as 95% CIs. As feasibility trials are not designed to establish efficacy, we will estimate the variance of outcome measures and calculate the effect size differences (with 95% CIs) on outcome measures from baseline to both follow-up points (on an intention-to-treat basis) in both groups. Where appropriate, the generalised estimating equation (GEE) will be employed to analyse the preliminary effects of BA across the two time points (baseline and 6 weeks). The GEE analysis will account for intra-correlated repeated outcome data and accommodate data missing at random.

**Research Team**

The research team has extensive experience in conducting clinical trials, patient public involvement, data management, clinical leadership within a custodial environment, and process observations. The Principal Investigator (PI) is Associate Professor Martin Jones, Project Director University of South Australia Department of Rural Health, Mount Barker, South Australia. PI MJ will be responsible for ensuring the trial adheres to the trial protocol. PI Steven Hutchinson, Nurse Consultant – Training & Practice Development, South Australia Prison Health Service will be responsible for satellite sites in Port Lincoln and the Riverland. PI Steven Hutchinson will in addition be responsible for ensuring the trial adheres to the trial protocol. Professor Richard Gray, Theme Lead, Healthy People Families and Communities La Trobe University, Victoria has 25 years of experience in conducting clinical trials. CI RG will provide advice regarding the delivery of a clinical trial and feasibility study. CI Kuan Tan will be the data custodian with responsibility for the secure storage of data. CI Pascal Dettwiller is employed by the SA Regional Clinical Trials Co-ordinating Centre, Office for Research, Department for Health, and Wellbeing, South Australia whose role will be to ensure we adhere to the principles of the tele-trial scheme. CI Dr. Thomas Turnbull, Medical Director, South Australia Prison Health Service, SA Health Central Adelaide Local Health Network, South Australia will provide medical leadership. CI Lucy Howard, Nurse Unit Manager, South Australia Prison Health Service, will be responsible for the Murray Bridge site. CI Jo Andrews, Nurse Unit Manager, South Australia Prison Health Service, will be responsible for the Port Augusta site. CI Shaun Dennis, Senior Mental Health Project Officer, Rural and Remote Mental Health Service, Barossa Hills Fleurier Local Health Network, South Australia will support the custodial health nurses to practice BA and provide remote supervision. CI Professor Dan Bressington, Professor of Nursing, Charles Darwin University, Northern Territory, has extensive experience in conducting clinical trials to examine the effectiveness of psychosocial interventions for people with serious mental health problems. CI DB will provide additional advice regarding the delivery of a clinical trial evaluating the feasibility of a psychosocial intervention. CI Dr. Shyamsundar Muthuramalingam, Consumer Engagement, SA Prison Health Service, SA Health Central Adelaide Local Health Network, South Australia, is the consumer engagement for SAPHS and ensures active consumer engagement at all stages of the trial. CI Sandra Walsh Research Assistant, University of South Australia Department of Rural Health, Whyalla, South Australia, will lead in conducting the interviews with the custodial health nurses and people in custody. CI Kuda Muyambi, University of South Australia Department of Rural Health, Mount Barker, South Australia, will support the ethics process and support CI SW in completing the interviews.

**Dissemination**

We will report findings from the present study using the Consolidated Standards of Reporting Trials (CONSORT) framework for reporting randomised pilot and feasibility trials (31). We intend to publish the findings in peer-reviewed journals. Authorship of any publications will be determined using the recommendations of the International Committee of Medical Journal Editors concerning authorship (32). Findings will also be presented at relevant seminars and professional conferences. Additionally, the participating prison sites will be provided with a summary of the findings for distribution to participants and relevant stakeholders. Public access to the study protocol will be ensured via the publication of the protocol in a professional peer-reviewed journal and registration of the trial protocol in the Australian New Zealand Clinical Trials Registry (ANZCTR) (33).

**Ethical considerations**

Ethical approval for the study will be sought from the Central Adelaide Local Health Network and the University of South Australia Human Research Ethics Committees. The study will be informed by the principles of Good Clinical Practice (GCP) enshrined in the “Declaration of Helsinki”, the Australian Code for the Responsible Conduct of Research, and the Australian Clinical Trial Handbook (34-36). The study may establish the feasibility of preparing custodial health nurses to deliver BA to people in custody with depression. If feasible, this will allow the research team to conduct an effectiveness study to assess if BA is clinically effective for people in custody with depression. The effectiveness study will also include a cost-benefit analysis. The outcomes of this program of work may increase access to an evidenced-based psychological treatment for depression to support people in custody. The benefits for the custodial health nurses include access to support and supervision from the BA experts which will enhance skills development. For the people in custody with depressive symptoms, the anticipated benefits include access to alternative psychotherapy which may reduce depressive symptoms.

Potential participants will be aware that their participation is entirely voluntary and that they may withdraw from the study at any point. Recruitment will be conducted by the tele-trial research nurse not working at the service provider to minimise the risk that people in custody may feel coerced to take part. People in custody will be given the opportunity, if required, to discuss the study with others who are important to them before consenting to take part in the trial. The potential benefits and risks of participation in the trial will be explained in Participant Information Sheets. Discussion of depression usually evokes emotive feelings. Participants will be provided with information about how they can access support.

The privacy of people in custody will be managed in a manner consistent with existing SA Prison Health Service arrangements. The intervention will be delivered at the prison health care centres and unless the prisoner discloses to other prisoners that they are attending the health centre for behavioural activation, the other prisoners will not be aware.

BA will be delivered by the custodial health nurses who will be responsible for recognising, responding, and reporting any distress arising from the intervention. The custodial health nurses will receive ongoing clinical supervision from telehealth BA experts who will have adequate professional indemnity insurance. After each prisoner has completed each session of BA, the custodial health nurses will ask them about their emotional well-being and if they have experienced any distress. If distress is experienced, the people in custody will have access to ongoing support through their existing support team and will be aware of how to contact this team. We have developed a distress protocol for people in custody (Attachment XIX). Any modifications to the protocol will be advised to the ethics committee and the Australian New Zealand Clinical Trials Registry (ANZCTR) (33), accordingly.

**Patient and public involvement**

An advisory committee will be established to develop, refine, and review the study recruitment materials. This will comprise prison advocacy groups from the four sites. We will ask the prison advocates for advice on interpreting the interviews we conduct with people in custody and advice on how best to share the findings with other patient public involvement groups who support people in custody.

**Safety considerations**

As noted, BA has not been conducted in SA prisons before. Thus, our safety considerations have been informed by other studies conducting similar interventions in community dwellings. An RCT study investigating the cost-effectiveness of BA compared with CBT for adults with depression reported that depression-related, but not treatment-related, serious adverse events occurred in three participants in the BA group and eight participants in the CBT group (37). The workers who delivered BA in the study were not specialist mental health workers, but university graduates who completed a five-day training program in BA.

To mitigate the risk of adverse effects we will put in place several procedures. We will ensure that custodial health nurses engage the people in custody at each assessment or the start of each BA session to check how they are feeling and if they experienced any unexpected effects of the intervention.

Custodial health nurses may find it distressing to work with people in custody with depression. Opportunities will be provided for the custodial health nurses to debrief with the BA experts via the telehealth supervision and support structure. In addition, all custodial health nurses will be invited to attend monthly supervision sessions with the BA experts. See also the Distress Protocol for custodial health nurses (Attachment XIX).

We will report all adverse events to the trial steering committee. If the trial steering committee considered that adverse events are a result of the intervention, we will suspend the trial. Adverse events will be analysed and where related to delivery mechanisms, will inform modifications to future trials.

**Potential Conflict of Interest**

Potential conflict of interest could arise from a preference for positive outcomes. This includes the custodial health nurses being invested in positive outcomes for their patients, and investigators being invested in a positive outcome of the trial. To minimise these potential conflicts of interest, custodial health nurses will be supervised to ensure fidelity to the treatment. The trial team includes members who are not familiar with behavioural activation and have no preconceptions regarding potential outcomes (including benefits) of the treatment. In addition, the establishment of the trial steering group will ensure that the research team is held to account for meeting the trial objectives, monitoring possible breaches, and reporting any safety concerns.

**Tele-trial Steering Group**

We will set up a Tele-trial Steering Group (TSG) comprising the research team, two prison advocates, and two independent members to provide safety oversight on the tele-trial processes and to review outcome data and any adverse events relating to the trial. The TSG will meet at least twice per year for the duration of the tele-trial.

**Indemnity and Insurance**

The indemnity and insurance covering the trial will be provided by the University of South Australia.

**Anticipated timeline**

It is anticipated that the online BA training program will commence in July 2023 and finish after 12 weeks in October 2023. The delivery of BA is expected to commence in January 2024. Recruitment will occur from November until December 2023 to be followed immediately afterward by the collection of baseline data. Follow-up will occur at 6 weeks and again at 3 months post-intervention. The tele-trial will finish in June 2024.

**Schedule of training and clinical trial enrolment, intervention, and assessments**

| **TRAINING PERIOD** | | | | | | | | | | | |
| --- | --- | --- | --- | --- | --- | --- | --- | --- | --- | --- | --- |
| 10-week online BA training program | Start: July 2023 | | | | | Finish: June 2024 | | | | | |
| **CLINICAL TRIAL PERIOD** | | | | | | | | | | | |
|  | Enrolment | Allocation | Post-allocation | | | | | | | | Close-out |
| Timepoint |  |  | Wk 1 | Wk 2 | Wk 3 | | Wk 4 | Wk 5 | Wk 6 | 3 months |  |
| **Enrolment**: |  |  |  |  |  | |  |  |  |  |  |
| Eligibility screening | **√** |  |  |  |  | |  |  |  |  |  |
| Informed consent | **√** |  |  |  |  | |  |  |  |  |  |
| Other procedures |  |  |  |  |  | |  |  |  |  |  |
| Allocation |  | **√** |  |  |  | |  |  |  |  |  |
| **Interventions**: |  |  |  |  |  | |  |  |  |  |  |
| Intervention A |  |  | **√** | **√** | **√** | | **√** | **√** | **√** | **√** | **√** |
| Intervention B |  |  | **√** | **√** | **√** | | **√** | **√** | **√** | **√** | **√** |
| **Assessments**: |  |  |  |  |  | |  |  |  |  |  |
| Baseline |  | **√** |  |  |  | |  |  |  |  | **√** |
| 6 weeks |  |  |  |  |  | |  |  | **√** |  | **√** |
| 3 months |  |  |  |  |  | |  |  |  | **√** | **√** |

**DISCUSSION**

BA is an effective treatment for depression. However, we do not yet understand its potential to support people in custody with depression when custodial health nurses. An important first step is to understand if BA is acceptable and feasible. Our feasibility trial will be undertaken in partnership with CALHN. As far as we are aware, this trial will be the first to establish the acceptability and feasibility of tele-trial delivered BA for this population. The results from this proposed feasibility study will help us to design and conduct a definitive randomised controlled trial, if feasible. We will use data with the effect size alongside the effect sizes reported in other studies will help us to determine the sample size for a full-scale trial. The feasibility data relating to participants who withdraw from our study will help us to calculate the number of participants we need to recruit. Our data regarding the completion of the outcome measures and the interview data will help us understand the feasibility and acceptability of the treatment and the outcome measures.

A key advantage of using BA with the study population is that it is easy and cheap to implement by workers who are not specialist mental health workers such as psychologists and mental health nurses (37-39). This may provide opportunities for scaling up BA across prison sites in Australia and overseas to support people in custody living with depression.

**Strengths**

We have ensured that the tele-trial study design provides an additional level of assurance for the custodial health nurses to adhere to the BA intervention. The adoption of a hub and spoke approach will enable potential participants to be recruited, trained, and/or treated nearest to where they live and work. The tele-trial will promote collaborative research and networking between the university and CALHN. The tele-trial will build the capacity of the rural health workforce to deliver BA thereby increasing access to support for people in custody living with depression.

**Limitations**

We anticipate that the tele-trial may experience challenges reaching the target sample size within the anticipated timeframe, leading to possible project delays. Due to potential restrictions and challenges (e.g. lockdowns, staff shortages, limited access to therapy spaces) in people in custody accessing and attending BA sessions, the timeline for this trial will be flexible (40). For similar reasons, a flexible and pragmatic approach to data collection will be adopted.

**Conclusion**

Findings from this feasibility study will inform the need to conduct a definitive trial. The findings are expected to inform the design and implementation of definitive trials testing the efficacy of BA in treating depression in people in custody. The study will also enable us to understand if people in custody derive benefits from using BA to help them manage their depression. Potentially, the results from the feasibility trial will help to scale up access to treatment of depressive symptoms for people in custody who often experience an unmet need.

**Trial registration** (ACTRN12623000346673p)

**REFERENCES**

1. Fazel S, Hayes AJ, Bartellas K, Clerici M, Trestman R. Mental health of prisoners: prevalence, adverse outcomes, and interventions. Lancet Psychiatry. 2016;3(9):871-81.

2. Al-Rousan T, Rubenstein L, Sieleni B, Deol H, Wallace RB. Inside the nation’s largest mental health institution: a prevalence study in a state prison system. BMC Public Health. 2017;17(1):342.

3. Andreoli SB, dos Santos MM, Quintana MI, Ribeiro WS, Blay SL, Taborda JGV, et al. Prevalence of Mental Disorders among Prisoners in the State of Sao Paulo, Brazil. PLOS ONE. 2014;9(2):e88836.

4. Fazel S, Seewald K. Severe mental illness in 33,588 prisoners worldwide: systematic review and meta-regression analysis. Br J Psychiatry. 2012;200(5):364-73.

5. Lohner J, Konrad N. Risk factors for self‐injurious behaviour in custody: Problems of definition and prediction. International Journal of Prisoner Health. 2007;3(2):135-61.

6. Favril L, Yu R, Hawton K, Fazel S. Risk factors for self-harm in prison: a systematic review and meta-analysis. Lancet Psychiatry. 2020;7(8):682-91.

7. Pratt D, Appleby L, Piper M, Webb R, Shaw J. Suicide in recently released prisoners: a case-control study. Psychol Med. 2010;40(5):827-35.

8. Barrenger SL, Draine J, Angell B, Herman D. Reincarceration Risk Among Men with Mental Illnesses Leaving Prison: A Risk Environment Analysis. Community Mental Health Journal. 2017;53(8):883-92.

9. National Centre for Clinical Excellence. Depression in adults: treatment and management, NICE guideline [NG222]. 29 June 2022: National Institute for Health and Care Excellence (NICE),; 2022.

10. Phillips A. Prescribing in prison: complexities and considerations. Nursing Standard. 2014;28(21):46-50.

11. Yoon IA, Slade K, Fazel S. Outcomes of psychological therapies for prisoners with mental health problems: A systematic review and meta-analysis. J Consult Clin Psychol. 2017;85(8):783-802.

12. Amoke CV, Ede MO, Nwokeoma BN, Onah SO, Ikechukwu-Ilomuanya AB, Albi-Oparaocha FC, et al. Effects of group cognitive-behavioral therapy on psychological distress of awaiting-trial prison inmates. Medicine (Baltimore). 2020;99(17):e18034.

13. Richards DA, Ekers D, McMillan D, Taylor RS, Byford S, Warren FC, et al. Cost and Outcome of Behavioural Activation versus Cognitive Behavioural Therapy for Depression (COBRA): a randomised, controlled, non-inferiority trial. The Lancet. 2016;388(10047):871-80.

14. Martell CR, Dimidjian S, Herman-Dunn R. Behavioral activation for depression: A clinician's guide: Guilford Publications; 2021.

15. Kanter JW, Manos RC, Bowe WM, Baruch DE, Busch AM, Rusch LC. What is behavioral activation? A review of the empirical literature. Clin Psychol Rev. 2010;30(6):608-20.

16. Chan AT, Sun GY, Tam WW, Tsoi KK, Wong SY. The effectiveness of group-based behavioral activation in the treatment of depression: An updated meta-analysis of randomized controlled trial. Journal of Affective Disorders. 2016;208:345-54.

17. Schulz KF, Altman DG, Moher D. CONSORT 2010 Statement: Updated guidelines for reporting parallel group randomised trials. J Clin Epidemiol. 2010;63(8):834-40.

18. Tong A, Sainsbury P, Craig J. Consolidated criteria for reporting qualitative research (COREQ): a 32-item checklist for interviews and focus groups. International Journal for Quality in Health Care. 2007;19(6):349-57.

19. Kroenke K, Spitzer RL, Williams JBW. The PHQ family of measures. Psychiatric Annals 2002;32:509-21.

20. Billingham SAM, Whitehead AL, Julious SA. An audit of sample sizes for pilot and feasibility trials being undertaken in the United Kingdom registered in the United Kingdom Clinical Research Network database. BMC Medical Research Methodology. 2013;13(1):104.

21. Costantini L, Pasquarella C, Odone A, Colucci ME, Costanza A, Serafini G, et al. Screening for depression in primary care with Patient Health Questionnaire-9 (PHQ-9): A systematic review. Journal of affective disorders. 2021;279:473-83.

22. Kroenke K, Spitzer RL. The PHQ-9: a new depression diagnostic and severity measure. Slack Incorporated Thorofare, NJ; 2002. p. 509-15.

23. Gadermann AM, Engel CC, Naifeh JA, Nock MK, Petukhova M, Santiago PN, et al. Prevalence of DSM-IV major depression among US military personnel: meta-analysis and simulation. Military medicine. 2012;177(suppl_8):47-59.

24. Pettersson A, Boström KB, Gustavsson P, Ekselius L. Which instruments to support diagnosis of depression have sufficient accuracy? A systematic review. Nordic journal of psychiatry. 2015;69(7):497-508.

25. Lins L, Carvalho FM. SF-36 total score as a single measure of health-related quality of life: Scoping review. SAGE Open Med. 2016;4:2050312116671725-.

26. The National Health and Medical Research Council. National Statement on Ethical Conduct in Human Research 2007 (Updated 2018) In: Australian Research Council and Universities Australia, editor. Canberra: Commonwealth of Australia; 2007.

27. Braun V, Clarke V. Using thematic analysis in psychology. Qualitative Research in Psychology. 2006;3(2):77-101.

28. QSR International Pty Ltd. NVivo (Version 12). 2020.

29. Microsoft Corporation. Microsoft Excel version 2022. 2022.

30. IBM. IBM SPSS Statistics 26.0. 2022.

31. Eldridge SM, Chan CL, Campbell MJ, Bond CM, Hopewell S, Thabane L, et al. CONSORT 2010 statement: extension to randomised pilot and feasibility trials. bmj. 2016;355.

32. Kornhaber RA, McLean LM, Baber RJ. Ongoing ethical issues concerning authorship in biomedical journals: an integrative review. Int J Nanomedicine. 2015;10:4837-46.

33. Askie LM. Australian New Zealand Clinical Trials Registry: history and growth. Journal of Evidence-Based Medicine. 2011;4(3):185-7.

34. World Medical Association. World Medical Association Declaration of Helsinki: Ethical Principles for Medical Research Involving Human Subjects. JAMA. 2013;310(20):2191-4.

35. Commonwealth of Australia. Australian clinical trial handbook: Guidance on conducting clinical trials in Australia using ‘unapproved’ therapeutic goods. In: Department of Health, editor.: Therapeutic Goods Administration; 2021.

36. National Health and Medical Research Council. Australian Code for the Responsible Conduct of Research 2018. In: Australian Research Council and Universities Australia, editor. Canberra: Commonwealth of Australia; 2018.

37. Richards DA, Ekers D, McMillan D, Taylor RS, Byford S, Warren FC, et al. Cost and Outcome of Behavioural Activation versus Cognitive Behavioural Therapy for Depression (COBRA): a randomised, controlled, non-inferiority trial. Lancet. 2016;388(10047):871-80.

38. Ekers D, Richards D, McMillan D, Bland JM, Gilbody S. Behavioural activation delivered by the non-specialist: phase II randomised controlled trial. British Journal of Psychiatry. 2011;198(1):66-72.

39. Ekers D, Webster L, van Straten A, Cuijpers P, Richards D, Gilbody S. Behavioural activation for depression: an update of meta-analysis of effectiveness and sub group analysis. PLoS One. 2014;9(6):e100100-e.

40. Pratt D, Gooding P, Awenat Y, Eccles S, Tarrier N. Cognitive Behavioural Suicide Prevention for Male Prisoners: Case examples. Cogn Behav Pract. 2016;23(4):485-501.
