# Supplementary material for: Behavioural activation for people in custody with depression: A protocol for a feasibility randomised controlled study
Source: PLoS One. 2024 Jun 13;19(6):e0304767. doi: 10.1371/journal.pone.0304767 (PMC11175500; doi:10.1371/journal.pone.0304767)
Supplement: S2 File — (DOCX) [file pone.0304767.s003.docx]

Supplementary file. Session guide for custodial nurses

Below is a suggested template for 12 conversations using Behavioural Activation. While this may be helpful for structuring your session, feel free to adjust it as needed clinically or to pursue therapeutic opportunities (such as a consumer progresses quickly or an opportunity arises to discuss values).

| **Conversation** | **Session structure/outline** | **Notes** |
| --- | --- | --- |
| **1** | - Ten Steps - Introduce BA - Explore potential benefits - Overview and expectations, i.e. Regular assessments, PHQ 2, BADS SF, Mood Monitoring, Activity Scheduling, Homework - Homework Task, complete PHQ 2 and BADS SF | Introduce the PHQ and BADS – explain what they do/don’t do and why client needs to complete.  PHQ2 and BADS – either as homework or before the next session (in the waiting room) – noting that some consumers will require support.  Explain role of BA is to break cycle of depression by trying new activities.  Describe your role in supporting client to become more engaged in activities.  Seek feedback. |
| **2** | - Ten Steps - Review of PHQ 2 (What this may mean) - Review of BADS SF (What this may mean, activating behaviors, avoidance behaviors) - Introduce Mood Diary - Homework complete Mood Diary | Explain how scores are used to track not diagnose.  PHQ and BADS as homework each week? |
| **3** | - Ten Steps - Review PHQ 2 - Review BADS SF - Review Mood Diary (explore fluctuations in mood, identify impact of different behaviors on mood) - Introduce Activity Scheduling - Homework Task Track activity for the week with your Mood Diary | The aim with the homework is to get a baseline level of activity, so we can see their current routine. |
| **4** | - Ten Steps - Review PHQ 2 - Review BADS SF - Review Mood Diary and Activity Schedule - Update Activity Schedule - Homework Activity Schedule and Mood Diary | Discuss current routine with mood, build case for change.  Talk about doing activities even when not motivated.  Build activity around routine, pleasure accomplishment.  Dealing with setbacks, barriers to activities.  Renegotiate alternative activities. |
| **5** | - Ten Steps - Review PHQ 2 - Review BADS SF - Review Mood Diary and Activity Schedule - Introduce TRAP and TRAC - Update Activity Schedule - Homework Activity Schedule and Mood Diary | While reviewing Mood Diary and Activity Schedule, notice avoidance and rumination – discuss TRAP and TRAC.  Talk about how rumination/avoidance understandable but can make things worse.  Talk about broad spectrum of rumination/avoidance behaviors. |
| **6** | - Ten Steps - Review PHQ 2 - Review BADS SF - Review Mood Diary and Activity Schedule - Introduce Values - Update Activity Schedule and adapts to persons values - Homework Activity Schedule and Mood Diary | Values conversation two purposes, building case for change and psychoeducation about values and mood (i.e. doing something you don’t like for someone you care about). |
| **7** | - Ten Steps - Review PHQ 2 - Review BADS SF - Review Mood Diary and Activity Schedule - Update Activity Schedule and adapts to persons values - Adjust activity to optimise routine - Homework Activity Schedule and Mood Diary | Psychoeducation regarding essential tasks that have a negative impact on mood – explore moving to another time. Scheduling positive activities before and after.  Doesn’t always need to be pleasurable activities. |
| **8** | - Ten Steps - Review PHQ 2 - Review BADS SF - Review Mood Diary and Activity Schedule - Update Activity Schedule and adapts to persons values - Adjust activity to optimise routine - Homework Activity Schedule and Mood Diary | Dealing with setbacks |
| **9** | - Ten Steps - Review PHQ 2 - Review BADS SF - Review Mood Diary and Activity Schedule - Update Activity Schedule and adapts to persons values - Adjust activity to optimise routine - Homework Activity Schedule and Mood Diary |  |
| **10** | - Ten Steps - Review PHQ 2 - Review BADS SF - Review Mood Diary and Activity Schedule - Update Activity Schedule and adapts to persons values - Adjust activity to optimise routine - Homework Activity Schedule and Mood Diary |  |
| **11** | - Ten Steps - Review PHQ 2 - Review BADS SF - Review Mood Diary and Activity Schedule - Update Activity Schedule and adapts to persons values - Adjust activity to optimise routine - Homework Activity Schedule and Mood Diary |  |
| **12** | - Ten Steps - Relapse prevention - Stick with Mood Diary - Scheduling activity - Where to get help | Summarise progress, i.e. improvements in avoidance and rumination behaviors.  Identify future problems and how to overcome them.  What to do if you relapse. |
